# Parotid gland metastasis of lung cancer: a case report

**DOI:** 10.1186/1477-7819-12-119

**Published:** 2014-04-24

**Authors:** Shuang Shi, Qi-Gen Fang, Fa-Yu Liu, Chang-Fu Sun

**Affiliations:** 1 Department of Pediatric Dentistry, School of Stomatology, China Medical University, No. 117 Nanjing North Street, Heping District, Shenyang, Liaoning 110002, People’s Republic of China; 2 Department of Oral and Maxillofacial Surgery, School of Stomatology, China Medical University, No. 117 Nanjing North Street, Heping District, Shenyang, Liaoning 110002, People’s Republic of China

**Keywords:** Parotid gland tumor, Small cell cancer, Lung cancer, Metastasis

## Abstract

**Background:**

Parotid gland metastasis in lung cancer is extremely rare, very few cases have been reported.

**Case presentation:**

We report on the case of a 61-year-old Chinese male patient who presented with parotid swelling metastasizing from advanced lung cancer. We therefore performed an operation of partial parotidectomy with preservation of the facial nerve and advised the patient receive chemotherapy, however, the patient died four months later.

**Conclusion:**

Although it is extremely rare, a potential metastasis of lung cancer should not be ignored in the diagnosis of parotid tumor. Preoperative routine examination, such as a chest X-ray and lung computational tomography scan, may play an important role in differential diagnosis. The management of the metastatic tumor to the parotid gland was controversial however, despite combined treatment modalities, long-term survival was not attained.

## Background

Lung cancer is a common tumor worldwide and frequently causes cancer-related deaths. It has a propensity to metastasize to any organ. To the best of our knowledge, the site of parotid gland in this case is very unusual and only a few articles have reported [1-5]. We describe a case of small-cell carcinoma of the lung which spread to the parotid gland.

## Case presentation

**Figure 1:**
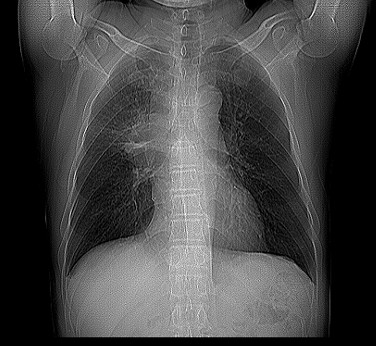
A high density shadow in the right hilar (X-ray).

**Figure 2:**
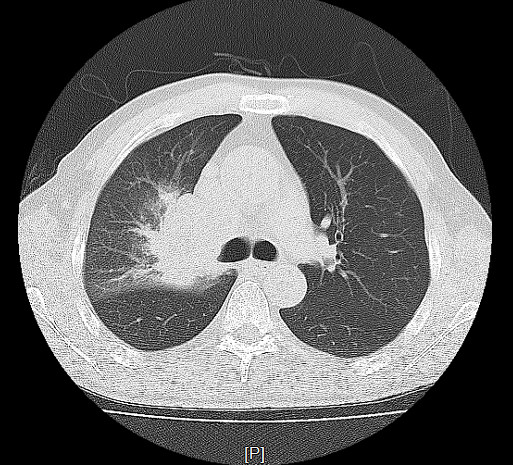
A shadow in the right upper lobe (Lung CT).

**Figure 3:**
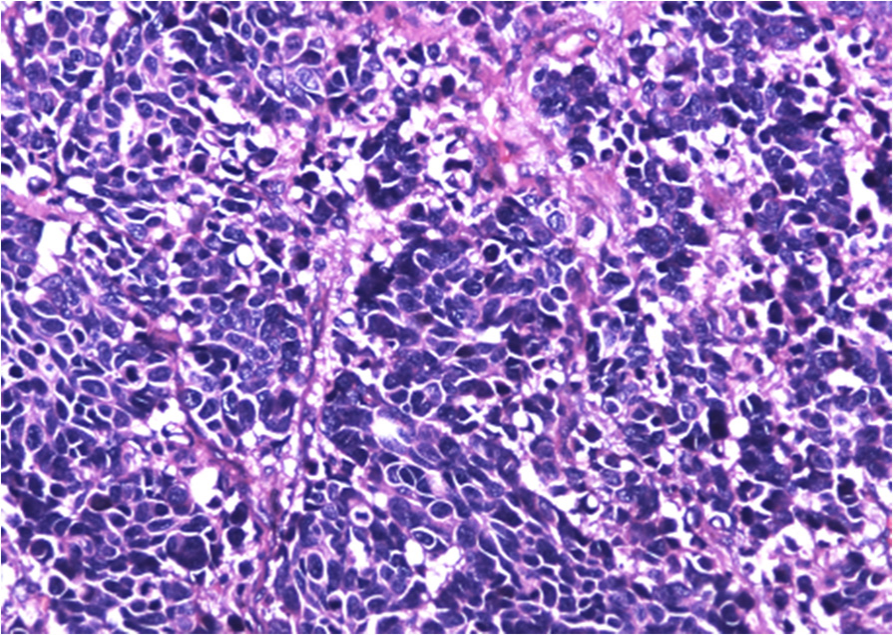
**Pathology: diffusely infiltrating atypical monotone cells in fibrous tissue.** (Hematoxylin and eosin, original magnification × 200).

A 61-year-old Chinese male presented with a progressive painful swelling in the right parotid gland for one month prior to attending our hospital. The patient had a history of heavy smoking, but did not complain of hemoptysis or other symptoms related to lung cancer. A clinical examination showed the size of the mass to be approximately 1 × 1 cm, with a good activity and a moderate texture. An ultrasound showed there to be a hypoechoic nodule with a size of 1.3× 1.3 × 0.9 cm. Therefore our initial diagnosis was that of a primary parotid tumor and we recommended surgical treatment. However, in preoperative routine examinations, a chest X-ray (Figure 1) showed there to be a high density shadow in the right hilar. At this stage we highly suspected that the mass was metastases and required further examination. A lung computational tomography (CT) scan (Figure 2) revealed a shadow in the right upper lobe, with a size of approximately 5.4 × 6.3 cm. The CT value was 40HU, and the enhanced CT value was 60HU, with multiple lymph nodes in the mediastinum appearing enlarged. After consulting with the patient, we performed an operation constituting of a partial parotidectomy and facial nerve dissection. Postoperative pathology reported a small-cell lung cancer metastases to parotid (Figure 3). Immunohistochemistry showed TTF(thyroid transcription factor)-1(+), Syn (Synaptophysin) (+), actin (-), S-100(-), P63 (-), EMA (epithelial membrane antigen) (-), ck(cytokeratin)20 (-). Therefore, we recommended that the patient receive postoperative radiation and chemotherapy.

## Discussion

Small-cell lung cancer comprises approximately 15% of bronchogenic carcinomas. It has a high malignancy potential and is usually associated with various paraneoplastic syndromes. At the time of diagnosis the disease is systemically disseminated in many cases, however, the diagnosis sometimes is made from a metastatic focus. The common site of distant spread is the mediastinal and supraclavicular lymph nodes, liver, bone, adrenals, and brain [5,6]. However, distant metastasis to the parotid gland is a very rare clinical event, to the best of our knowledge. The possible reasons for this may be the anatomical location of the parotid gland and that parenchymal metastasis occurred via hematogenous rather than by lymphatic spread.

As far as we are aware, it is a known fact that most of the metastasis to the submandibular gland are from a distant source, with much fewer being from the head and neck area, which is exactly the opposite when compared to the parotid gland. We hypothesize that the possible reasons for this situation are the difference in the anatomic relationships, number of the lymph nodes, and their drainage between the parotid gland and submandibular gland.

The treatment of metastatic parotid tumor is a matter of debate. Raut *et al.*[7] presented that, for most tumors restricted to the parotid, a total parotidectomy whilst protecting the facial nerve, followed by radiation therapy, should be the protocol. Nuyens *et al.*[8] found that the predominant amount of metastatic diseases to the parotid gland were squamous cell carcinomas and malignant melanomas, and concluded that a total or partial parotidectomy with consideration for neck dissection, followed by post-operation radiation, should be included in the treatment. Bumpous *et al. *[9] reviewed related cases and concluded that, when a positive lymph node was detected in the parotid, a combination of surgical management with postoperative irradiation could increase locoregional control. However, Jecker *et al.*[10] reported that radical parotid surgery had little effect on improving the life expectancy and concluded that all the current therapeutic procedures were ineffective. In our view, for parotid gland metastases from head and neck cancer origin, a total or partial parotidectomy with a neck dissection is acceptable because of the tendency of the disease to spread via the lymphatic system. For parotid gland metastases originating from a distant organ such as the lung, a neck dissection may be unnecessary because the metastasis is more likely to be caused by hematogenous. In this case, we performed an operation consisting of a partial parotidectomy with preservation of facial nerve, and advised that the patient receive chemotherapy based on the fact that small-cell lung cancer is sensitive to chemotherapy. The patient died four months later, the situation highlighted the fact that metastasis to the parotid gland represented an advanced stage of the disease with a subsequently poor prognosis [5].

## Conclusions

Although it is extremely rare, a potential metastasis of lung cancer should not be ignored in the diagnosis of a parotid tumor. Preoperative routine examinations such as a chest X-ray and lung CT, may play an important role in the differential diagnosis. The management of the metastatic tumor to the parotid gland was controversial, however, despite combined treatment modalities long-term survival remained unsuccessful.

## Consent

Written informed consent was obtained from the patient for the publication of this case report and any accompanying images. A copy of the written consent is available for review by the Editor of this journal.

## Abbreviations

CT: computation tomography; TTF: thyroid transcription factor; Syn: Synaptophysin; EMA: epithelial membrane antigen; Ck: cytokeratin.

## Competing interests

The authors declare that they have no competing interests.

## Authors’ contributions

Q-GF and SS contributed to the conception, design, and acquisition of information and write this paper; F-YL and C-FS helped to draft the manuscript and revise the paper. All authors read and approved the final manuscript.
